# Identification of CALU and PALLD as Potential Biomarkers Associated With Immune Infiltration in Heart Failure

**DOI:** 10.3389/fcvm.2021.774755

**Published:** 2021-12-01

**Authors:** Xing Liu, Shiyue Xu, Ying Li, Qian Chen, Yuanyuan Zhang, Long Peng

**Affiliations:** ^1^Department of Cardiovascular Medicine, The Third Affiliated Hospital, Sun Yat-sen University, Guangzhou, China; ^2^Department of Hypertension and Vascular Disease, The First Affiliated Hospital, Sun Yat-sen University, Guangzhou, China; ^3^Department of Dermatology, Guangzhou Eighth People's Hospital, Guangzhou Medical University, Guangzhou, China

**Keywords:** heart failure, ischemic cardiomyopathy, inflammatory activation, biomarker, diagnosis

## Abstract

**Background:** Inflammatory activation and immune infiltration play important roles in the pathologic process of heart failure (HF). The current study is designed to investigate the immune infiltration and identify related biomarkers in heart failure patients due to ischemic cardiomyopathy.

**Methods:** Expression data of HF due to ischemic cardiomyopathy (CM) samples and non-heart failure (NF) samples were downloaded from gene expression omnibus (GEO) database. Differentially expressed genes (DEGs) between CM and NF samples were identified. Single sample gene set enrichment analysis (ssGSEA) was performed to explore the landscape of immune infiltration. Weighted gene co-expression network analysis (WGCNA) was applied to screen the most relevant module associated with immune infiltration. The diagnostic values of candidate genes were evaluated by receiver operating curves (ROC) curves. The mRNA levels of potential biomarkers in the peripheral blood mononuclear cells (PBMCs) isolated from 10 CM patients and 10 NF patients were analyzed to further assess their diagnostic values.

**Results:** A total of 224 DEGs were identified between CM and NF samples in GSE5406, which are mainly enriched in the protein processing and extracellular matrix related biological processes and pathways. The result of ssGSEA showed that the abundance of dendritic cells (DC), mast cells, natural killer (NK) CD56dim cells, T cells, T follicular helper cells (Tfh), gammadelta T cells (Tgd) and T helper 2 (Th2) cells were significantly higher, while the infiltration of eosinophils and central memory T cells (Tcm) were lower in CM samples compared to NF ones. Correlation analysis revealed that Calumenin (CALU) and palladin (PALLD) were negatively correlated with the abundance of DC, NK CD56dim cells, T cells, Tfh, Tgd and Th2 cells, but positively correlated with the level of Tcm. More importantly, CALU and PALLD were significantly lower in PBMCs from CM patients compared to NF ones.

**Conclusion:** Our study revealed that CALU and PALLD are potential biomarkers associated with immune infiltration in heart failure due to ischemic cardiomyopathy.

## Introduction

Heart failure refers to the complex clinical syndrome and the end-stage manifestations of cardiovascular disease (1, 2). Large-scale epidemiological analysis shows that the global prevalence of HF is on the rise due to the aging of the population and the progress in the diagnosis and treatment of cardiovascular diseases. In developed countries, the prevalence of HF is 1.5–2.2% (3). The latest report in China shows that the prevalence of HF among residents ≥35 years old is 1.3%, that is, there are ~13.7 million patients (3).Therefore, HF has always been a hot spot in the field of cardiovascular research.

HF is caused by the complicated interaction of myocardial damage, neurohormonal activation, inflammatory response, and renal dysfunction (4–6). During the process, the cytoskeletal and membrane associated proteins are increased and disorganized, while the contractile myofilaments and sarcomeric proteins are decreased in the heart (7). In addition, cardiomyocytes in the failing heart display impaired excitation-contraction coupling due to the decreased calcium transients, enhanced diastolic sarcoplasmic reticulum (SR) calcium leak and diminished SR calcium sequestration (8). Although the pathogenesis of HF is still perplexing, the persistent inflammation and immune abnormalities are believed to participate in the pathogenesis across the spectrum of HF (9). Elevated and long-lasting leukocyte recruitment mediated by G protein-coupled receptor kinase 5 (GRK5) in the injured heart is reported to be associated with chronic cardiac inflammation and heart failure (10). Moreover, evidence indicates that transcriptome changes in immune cells could affect the prognosis of HF. DNA methyltransferase 3 alpha (DNMT3A) mutations in monocytes significantly increase the expression of inflammatory genes and are correlated with the aggravation of chronic HF (11). Metabolically active genes such as fatty acid binding protein 5 (FABP5) are highly enriched in classical monocytes from heart failure patients, whereas b-catenin expression was significantly higher in another functionally distinct monocyte subset (CD14^++^CD16^+^ intermediate monocytes) (12). These studies suggest that further understanding of the inflammatory response and immune cell infiltration in HF is of great significance for optimizing the diagnosis and treatment of heart failure.

In recent years, microarray technology and integrated bioinformatics analyses have been performed to identify novel genes related to various diseases that might act as diagnostic and prognostic biomarkers (13–15). However, the diagnostic value of genes associated with immune infiltration in heart failure remains unclear. Thus, in the current study, we downloaded two microarray datasets of HF from the GEO database and used bioinformatic methods to screen for immune infiltration related biomarkers in heart failure, and to provide a theoretical basis for the diagnosis and treatment of HF patients.

## Materials and Methods

### Ethics Statement

This study was approved by the institutional review board of the Third Affiliated Hospital, Sun Yat-sen University (IRB: 202102-201-01).

### Data Source

In the current study, gene expression profiles of 16 non-failure controls (NF) and 108 heart failure samples caused by ischemic cardiomyopathy (CM) in GSE5406 dataset and 14 NF and 13 CM samples in GSE116250 dataset were downloaded from GEO database.

### Identification and Functional Enrichment Analysis of DEGs

Limma R package was used to identify DEGs between NF and CM samples with |log2FC| >0.5 and adjusted *p* < 0.05 in GSE5406 datasets. ClusterProfiler R package was applied for GO and KEGG pathway enrichment analyses of DEGs. Biological process (BP), molecular function (MF) and cellular component (CC) were included in the GO analysis.

### ssGSEA

ssGSEA was performed by Gene Set Variation Analysis (GSVA) R package to analyze the infiltraion of 24 immune cells in NF and CM samples (16). The 24 immune cells were TFH, Th2 cells, B cells, T cells, Tgd, NK CD56dim cells, Tem, macrophages, neutrophils, Th1 cells, mast cells, cytotoxic cells, DC, iDC, eosinophils, T helper cells, aDC, TReg, pDC, NK CD56bright cells, NK cells, Th17 cells, CD8 T cells, and Tcm.

### WGCNA Analysis

A sample clustering tree map was first constructed to detect and eliminate outliers. Then, WGCNA was performed based on the gene expression profiles from GSE5406 dataset and sample traits (differentially infiltrated immune cells between NF and CM samples). The pick Soft Threshold function of WGCNA was used to calculate β from 1 to 20 in order to select the best soft threshold. Based on the selected soft threshold, the adjacency matrix was converted to topological overlap matrix to construct the network, and the gene dendrogram and module color were established by using the degree of dissimilarity. We further divided the initial module by dynamic tree cutting and merged similar modules. The Pearson correlation coefficient between the module eigengenes and sample traits were calculated to find out the most relevant module (hub module) associated with sample traits.

### Identification of Biomarkers in CM

First, DEGs were intersected with genes from the hub module in WGCNA analysis to obtain immune infiltration related candidate genes. Next, gene signature was selected by least absolute shrinkage and selection operator (LASSO) algorithm using glmnet R package (17) and support vector machine-recursive feature elimination (SVM-RFE) method using e1071 package (18), respectively. Robust gene signature was identified by overlapping gene signature obtained from LASSO and SVM-RFE. The diagnostic values of gene signature were evaluated by receiver operating curves (ROC) curves. Then, the external validation dataset GSE116250 was used to verify the expressions and diagnostic values of gene signature identified in GSE5406. Validated gene signature was identified as robust diagnostic biomarkers in heart failure.

### Functional Analysis of Biomarkers in CM

To investigate the potential mechanisms of diagnostic biomarkers in regulating heart failure, 108 patients in GSE5406 were divided into high- and low-expression groups based on the median expression of each diagnostic biomarker. Moreover, to explore the relationship between diagnostic biomarkers and immune infiltration, the correlations between the expressions of diagnostic biomarkers and the abundance of differentially infiltrated immune cells were calculated.

### Subject Characteristics and Realtime-PCR

Patients aged 18 and older, diagnosed with CHD by coronary computed tomography angiography or coronary angiography, with ejection fraction of 40% or less were enrolled into CM group. Age-matched CHD patients without heart failure (ejection fraction of 50% or above) were enrolled into NF control group. Patients with a history of malignancy, acute coronary syndrome, pulmonary embolism, renal failure [Glomerular filtration rate <60 ml/(min·1.73 m^2^)] were excluded. The characteristics of CM and NF patients were shown in Supplementary Table 3.

RNA of PBMCs from CM (*n* = 10, 7 male and 3 female) and NF (*n* = 10, 8 male and 2 female) patients were extracted using Nuclezol LS RNA Isolation Reagent (ABP Biosciences Inc.) according to manufacturer's instructions. Collected RNA was diluted using nuclease-free water and electrophoresed on a denaturing formaldehyde agarose gel to visualize rRNA and ensure overall sample quality. RNA concentrations and purity were detected on ultraviolet spectrophotometer (Jinghua, Shanghai, China). Reverse transcription was performed on 1 μg total RNA from each sample using the SureScript-First-strand-cDNA-synthesis-kit (GeneCopoeia) according to manufacturer's instructions, and a CFX96 Real-time PCR System (Bio-Rad) was utilized to conduct the real time quantitative PCR (qPCR) reactions. BlazeTaq™ SYBR® Green qPCR Mix 2.0 (GeneCopoeia) was used for qPCR reactions, using 4 μL cDNA and appropriate volumes of specific primers in a final 10 μL volume. Triplicate reactions were performed to ensure accuracy. Glyceraldehyde-3-phosphate dehydrogenase (GAPDH) was used as the reference gene, and the relative gene expression was quantified by the 2^−Δ*ΔCT*^ method (19). The primer sequences were given in Table 1.

**Table 1:** Primers sequence.

**Genes**	**Forward**	**Reverse**
CALU	GTTTCTTATGTGCCTGTCCCT	TTCCTTGCTCTCTTCTGGTGT
PALLD	GCCTACTTTCCTCCTGTTTTT	AGTGGTCATTGTTGGATTCTC
GAPDH	CGCTGAGTACGTCGTGGAGTC	GCTGATGATCTTGAGGCTGTTGTC

### Statistical Analysis

All data were analyzed by R software (version 4.0.0). Wilcoxon test was used to compare the data between two groups, and significant difference was considered as *p* < 0.05 unless specified.

## Results

### Transcriptome Profile Analyses of NF and CM Samples

**Figure 1:**
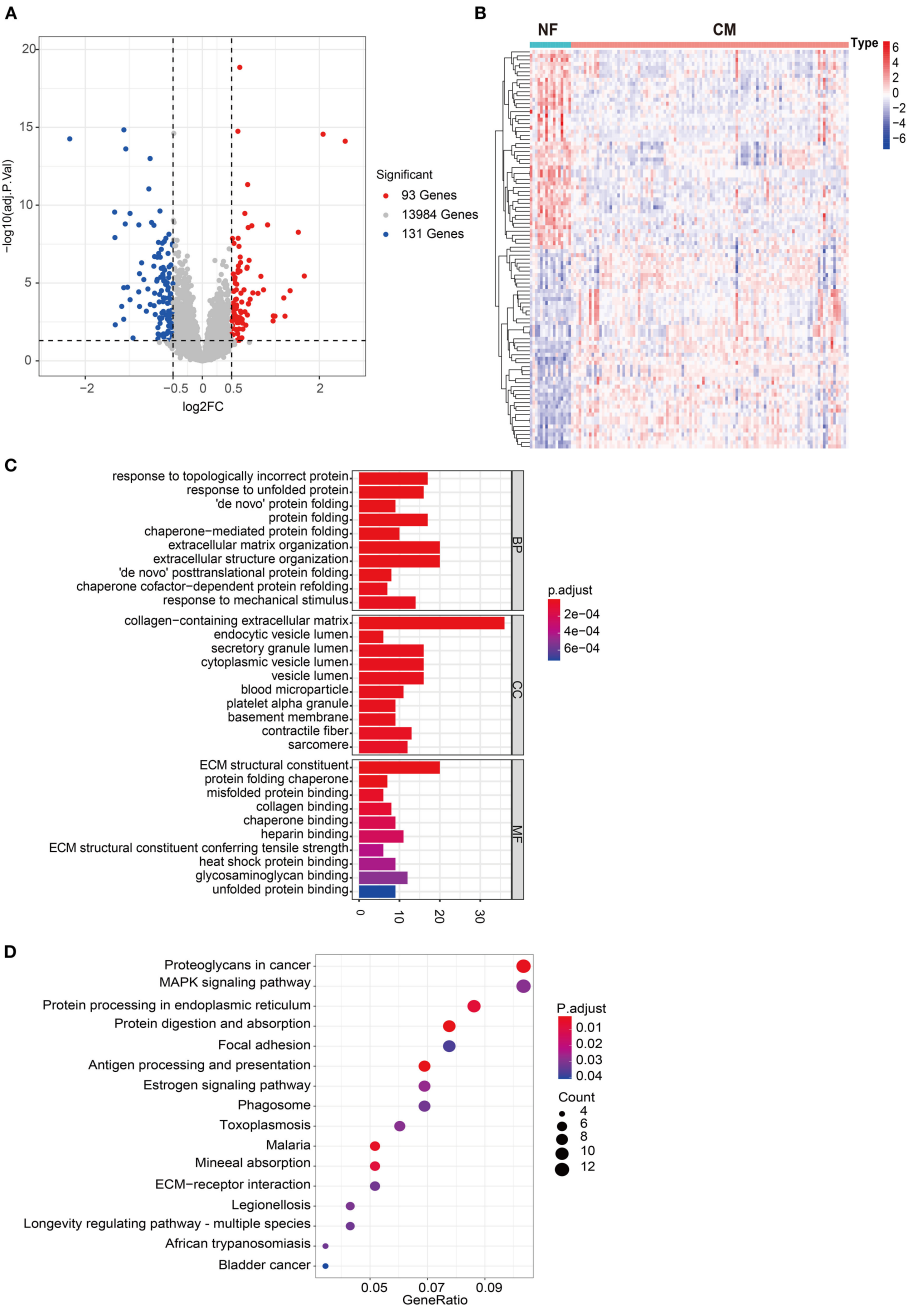
Comprehensive analyses of the transcriptome profiles of NF and CM samples. **(A)** Volcano plot of significant DEGs between NF and CM samples. **(B)** A heatmap of the top 50 significantly upregulated or downregulated DEGs. **(C)** Bar plot of top 10 enriched GO terms of DEGs in each category. BP, biological process; CC, cellular components; MF, molecular functions. **(D)** Bubble plot of significantly enriched KEGG pathways of DEGs.

A total of 224 DEGs were identified in GSE5406, including 93 up-regulated and 131 down-regulated genes in CM group compared to NF group (Figure 1A). The expression profile of top 50 up-regulated DEGs and top 50 down-regulated DEGs were shown in the heatmap (Figure 1B). To investigate the biological function of DEGs, we performed GO and KEGG pathway analysis. A total of 217 BP, 42 CC, 37 MF, and 16 KEGG pathways were significantly enriched (Supplementary Tables 1, 2). As shown in Figure 1C, DEGs were mainly enriched into protein processing and extracellular matrix (ECM) related BPs, including response to topologically incorrect protein, response to unfolded protein (UPR), “*de novo*” protein folding, protein folding, chaperone-mediated protein folding, extracellular matrix organization, extracellular structure organization, “*de novo*” posttranslational protein folding, chaperone cofactor-dependent protein refolding, response to mechanical stimulus. Consistent with the results of GO analysis, protein processing and ECM related pathways were significantly enriched, including protein processing in endoplasmic reticulum, ECM-receptor interaction, and focal adhesion. In addition, estrogen signaling and MAPK signaling pathways showed to have close relationship with heart failure (Figure 1D).

### Identification of Immune Infiltration Pattern in CM

**Figure 2:**
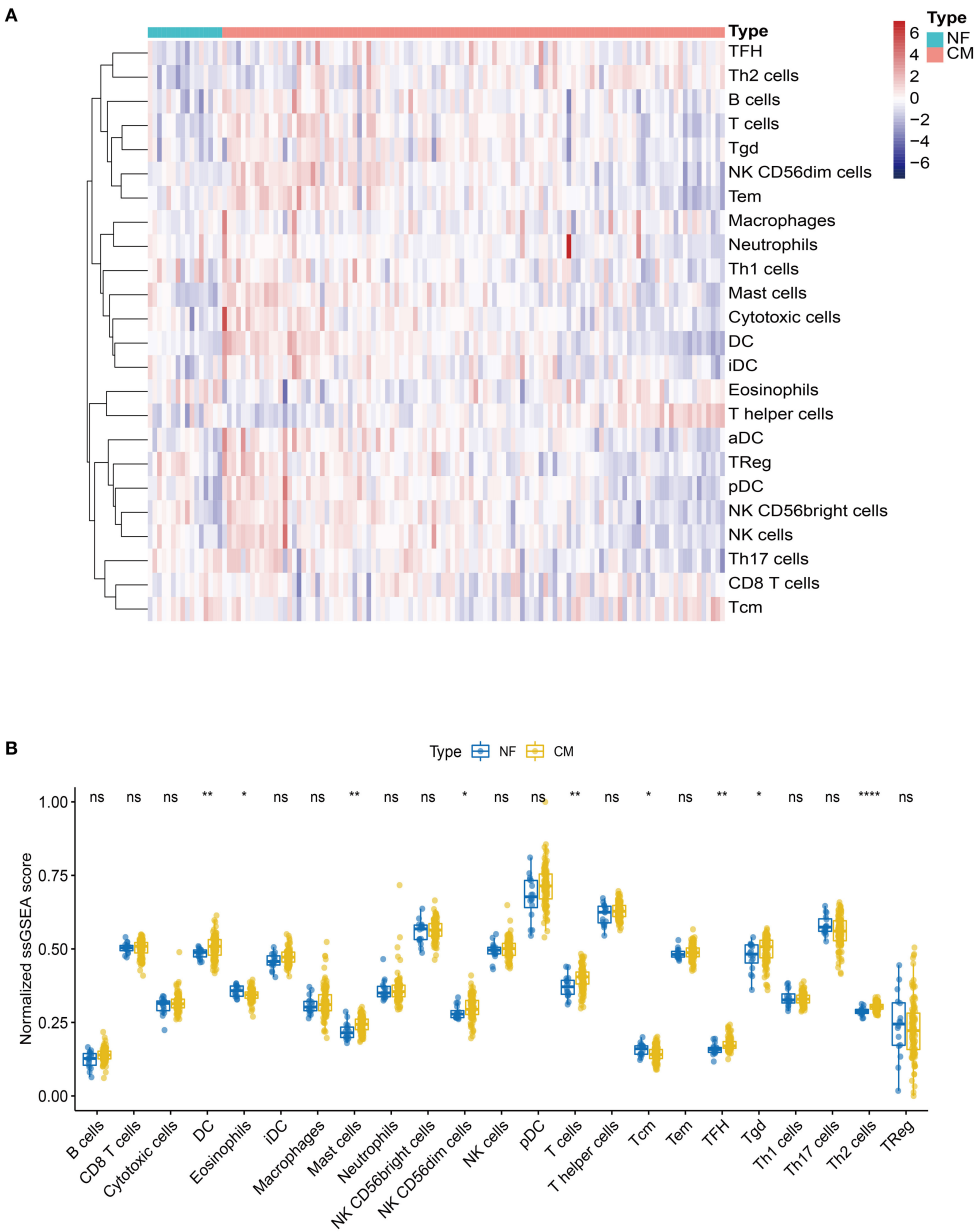
Landscape of the immune infiltration in NF and CM samples. **(A)** Heatmap of the immune infiltration profiles of NF and CM samples analyzed by ssGSEA score-based method. **(B)** Comparison of immune cell infiltration between NF and CM samples. *p < 0.05, **p < 0.01, ****p < 0.001.

Mounting evidence suggest that immune cells play important roles in heart failure (14–16). Thus, we explored the profile of immune cell infiltration in CM and NF samples by ssGSEA. Twenty-four subpopulations of infiltrated immune cells in CM and NF samples were identified and presented in the heatmap (Figure 2A). Interestingly, we found that the abundance of DC, mast cells, NK CD56dim cells, T cells, Tfh, Tgd, and Th2 cells were significantly higher, while the infiltration of eosinophils and Tcm were significantly lower in CM samples compared to NF ones (Figure 2B). These results indicate that the inflammatory response of these immune cells may be critical for the etiology of heart failure.

### Screening for Gene Signature of Immune Infiltration in CM

**Figure 3:**
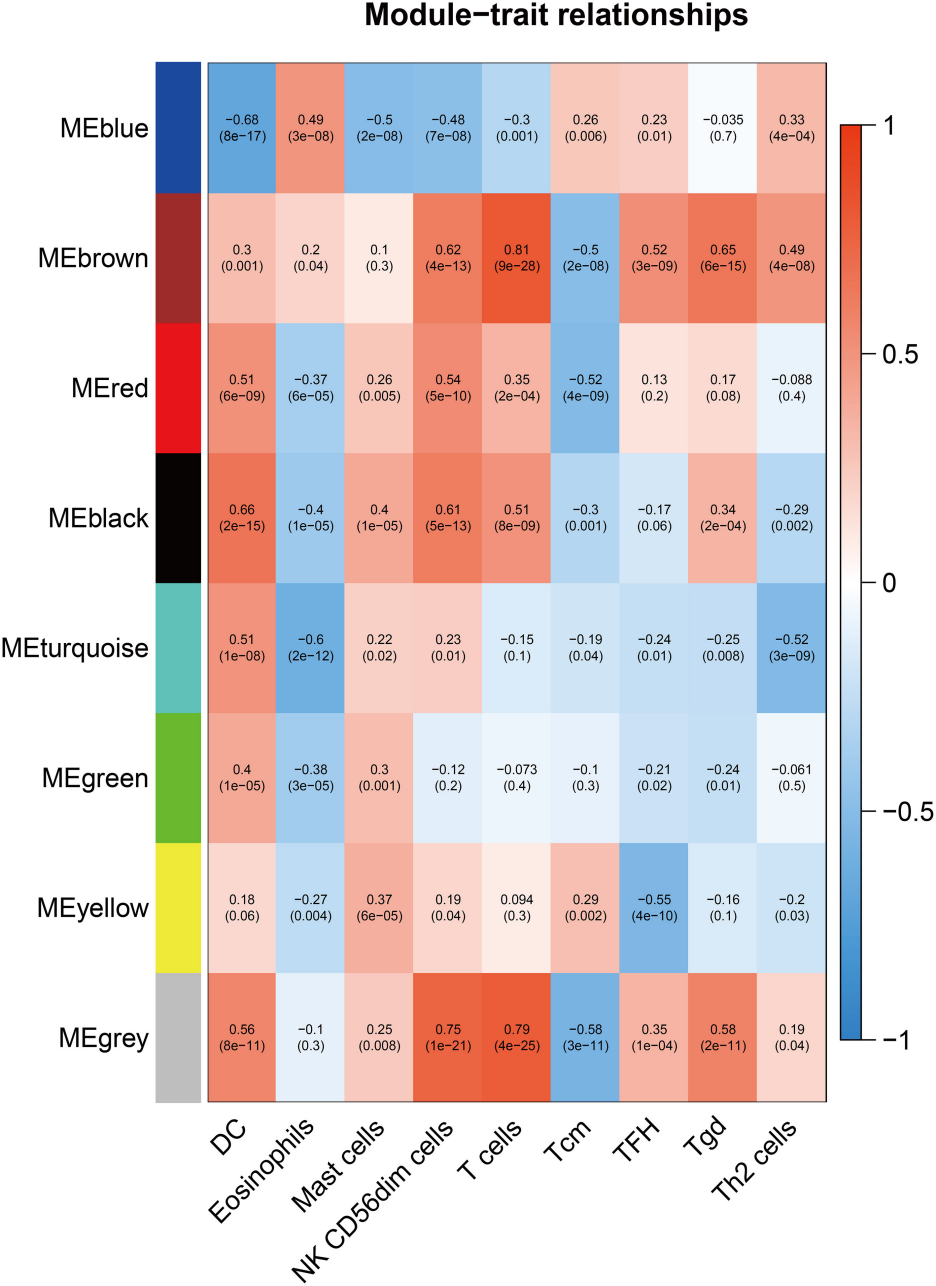
Screening of the hub module associated with immune infiltration in CM. Heatmap of the correlation between module eigengene and differentially infiltrated immune cells. Each row represents a color-coded module eigengene, each column represents a type of infiltrated immune cells. The number in each cell means the correlation coefficient and *p*-value.

To further explore the genes mostly correlated with the inflammatory response in CM, we performed WGCNA to screen for the hub module associated with above infiltrated immune cells. After eliminating the outlier samples (Supplementary Figure 1), we built the sample dendrogram and trait heatmap (Supplementary Figure 2). By using the pick Soft Threshold function of WGCNA, we found the optimal soft threshold power was 9, in which *R*^2^ was 0.85 (Supplementary Figure 3A). After merging similar modules, eight modules from the co-expression network were identified (Supplementary Figure 3B). According to the module-trait relationships in Figure 3, we found that the MEbrown module was the most relevant module associated with DC (Cor = 0.3, *p* < 0.01), NK CD56dim cells (Cor = 0.62, *p* < 0.01), T cells (Cor = 0.81, *p* < 0.01), Tcm (Cor = −0.5, *p* < 0.01), TFH (Cor = 0.52, *p* < 0.01), Tgd (Cor = 0.65, *p* < 0.01) and Th2 cells (Cor =0.49, *p* < 0.01). Thus, MEbrown module was selected for downstream analysis.

**Figure 4:**
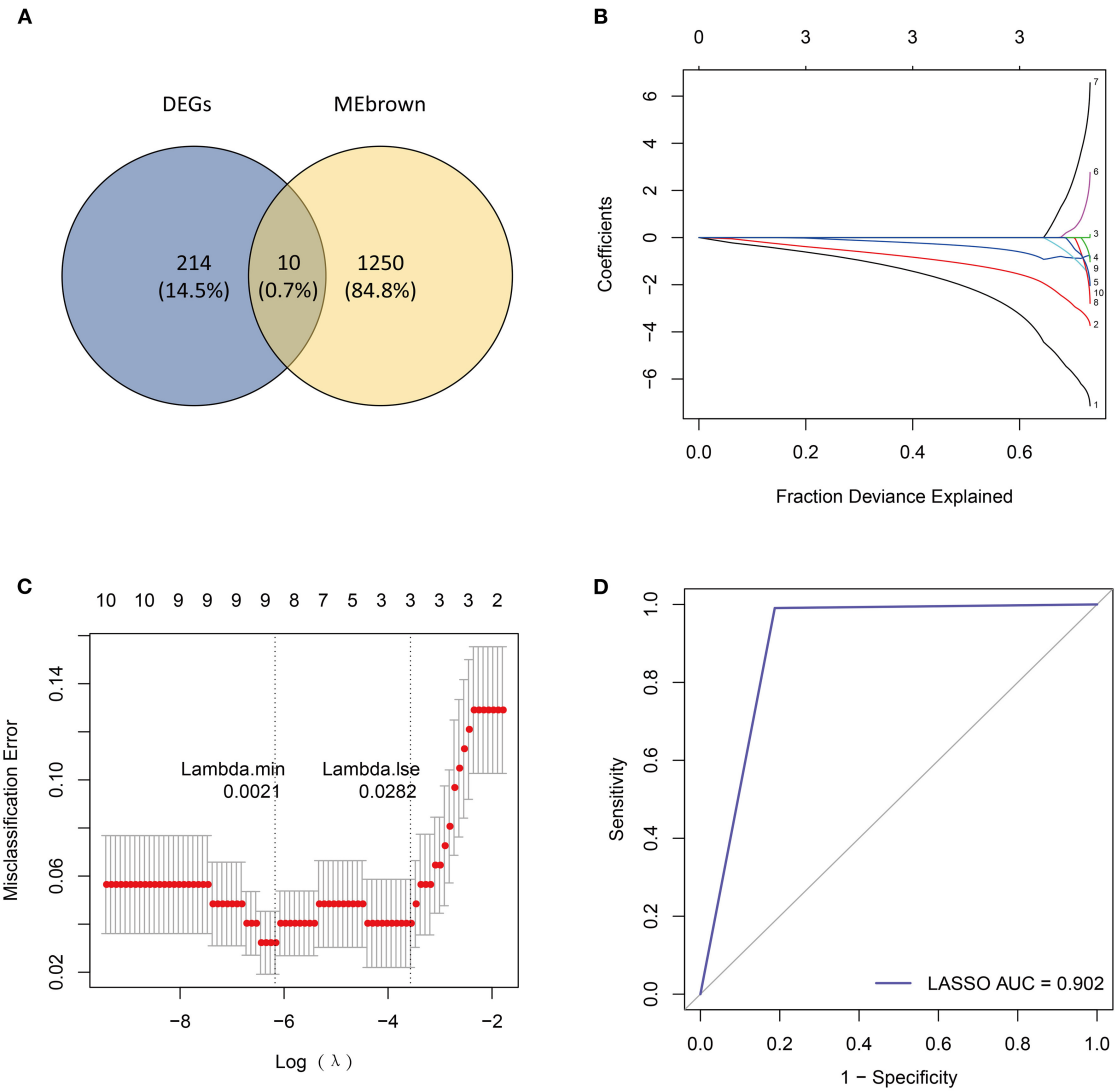
Identification of candidate genes by LASSO regression model. **(A)** Venn diagram of 10 overlapped candidate genes shared by DEGs and MEbrown module. **(B)** LASSO coefficient profiles of candidate genes. **(C)** Cross-validation to select the optimal tuning parameter log (Lambda) in LASSO regression analysis. **(D)** ROC curve evaluation of LASSO regression analysis.

**Figure 5:**
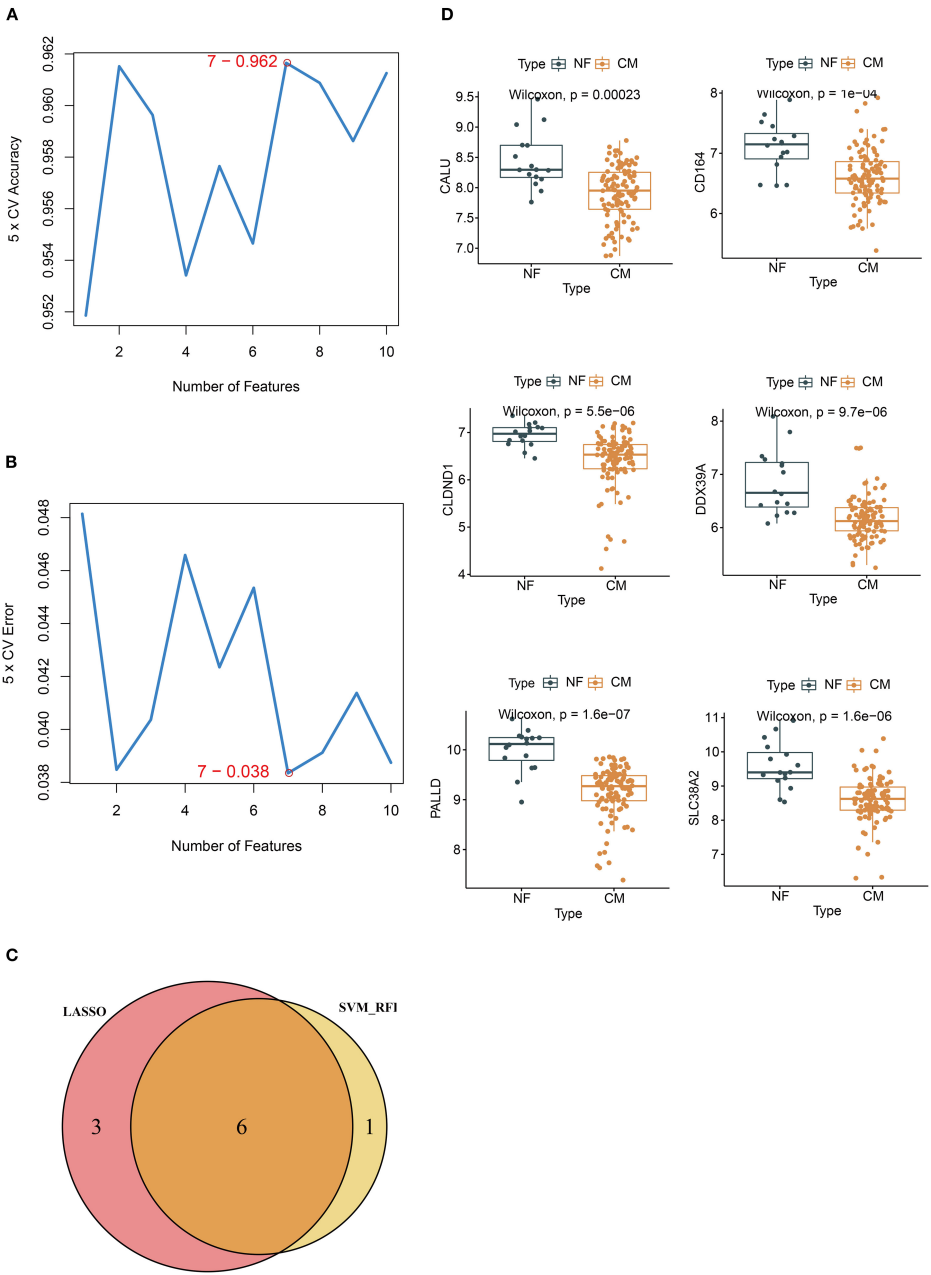
Identification of candidate genes by SVM-RFE. **(A)** 7 gene signatures are identified by SVM-RFE analysis with the accuracy of 0.962 and **(B)** error of 0.038. **(C)** Venn diagram of six overlapped candidate genes shared by the LASSO and SVM-RFE algorithms. **(D)** The expressions of candidate diagnostic biomarkers in the NF and CM samples from GSE5406 dataset.

Next, we overlapped DEGs with genes in the MEbrown module and obtained 10 candidate genes (Figure 4A). Ten candidate genes were input into LASSO and SVM-RFE to identify gene signature, respectively. LASSO identified nine gene signatures under lambda.min = 0.0021, including PALLD, DexD-box helicase 39A (DDX39A), stress induced phosphoprotein 1 (STIP1), solute carrier family 38 member 2 (SLC38A2), CALU, CD164 molecule (CD164), selenoprotein T (SELT), four and a half LIM domain 1 (FHL1) and claudin domain containing 1 (CLDND1) (Figures 4B,C). The accuracy of LASSO was evaluated by ROC curve that the area under the ROC curve (AUC) was 0.902 (Figure 4D). Meanwhile, we identified 7 gene signatures by SVM-RFE, including PALLD, DDX39A, CD164, CLDND1, SLC38A2, CALU, and heat shock protein family D member 1 (HSPD1) with the accuracy of 0.962 (Figures 5A,B). To get the robust gene signature in heart failure, we overlapped genes from LASSO and SVM-RFE and got six gene signatures (Figure 5C), including PALLD, DDX39A, CD164, CLDND1, SLC38A2, and CALU. The expression levels of PALLD, DDX39A, CD164, CLDND1, SLC38A2, and CALU were all significantly higher in NF samples compared to CM ones in GSE5406 (Figure 5D).

### Verification of the Potential Biomarkers for CM

**Figure 6:**
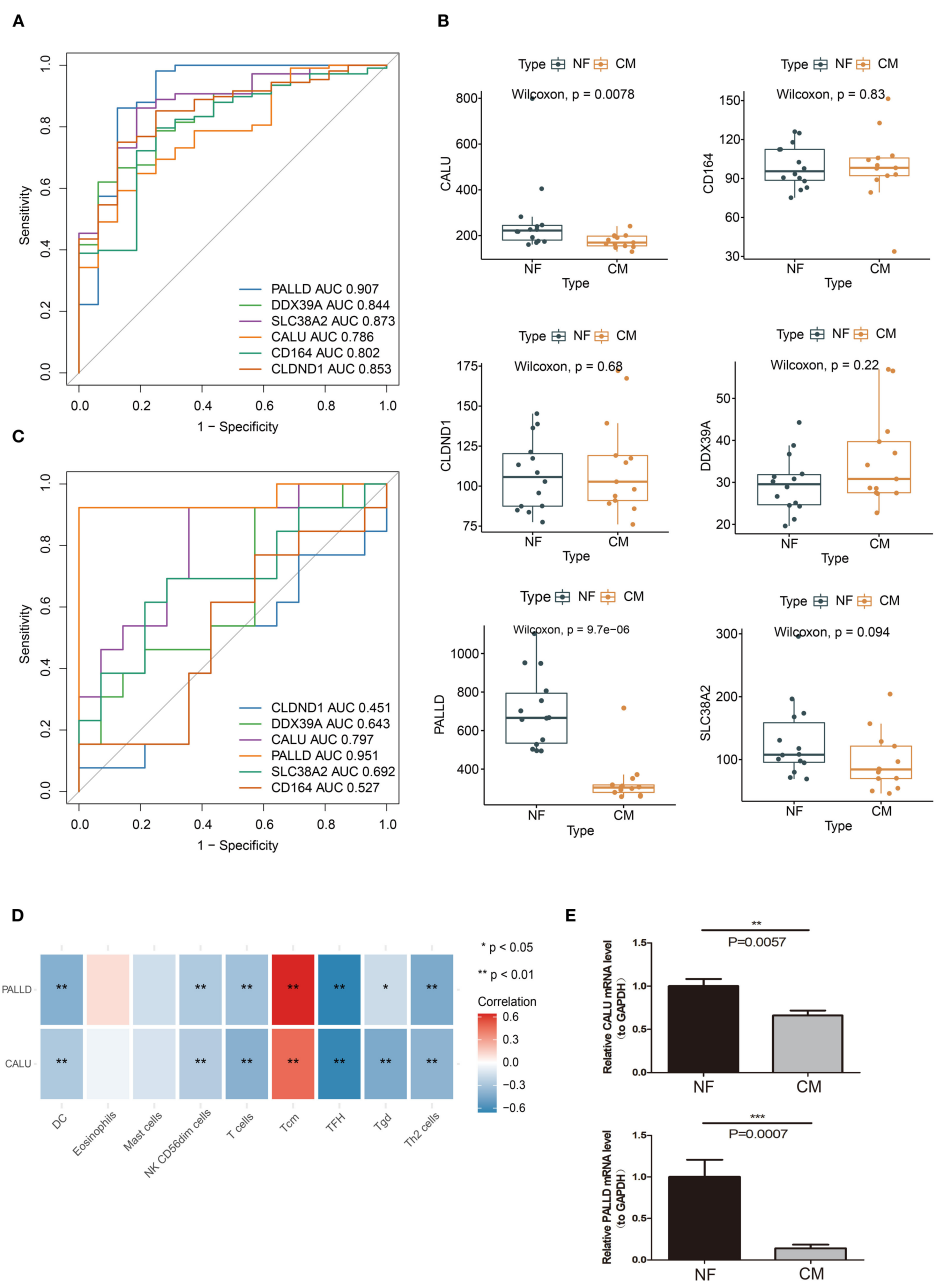
Verification of biomarkers for CM. **(A)** ROC curve evaluation of the diagnostic effectiveness of candidate biomarkers using GSE5406 dataset. **(B)** The expressions of candidate diagnostic biomarkers in the GSE116250 dataset. **(C)** ROC curve evaluation of the diagnostic effectiveness of candidate biomarkers using GSE116250 dataset. **(D)** Heatmap of correlations between PALLD, CALU and differentially infiltrated immune cells. **(E)** Real-time PCR analyses of the expression levels of PALLD and CALU in PBMCs isolated from CM and NF patients *p < 0.05, **p < 0.01, ***p < 0.001

We further evaluated the diagnostic values of PALLD, DDX39A, CD164, CLDND1, SLC38A2, and CALU in GSE5406 by ROC curves. We found that they all had high accuracy with AUC >0.7 (Figure 6A). To verify the diagnostic values of the candidate biomarkers, we examined their expressions in an external validation dataset GSE116250 to get robust diagnostic biomarkers. We found that the expression trends of CALU and PALLD in GSE116250 were consistent with those in GSE5406 (Figure 6B), and that CALU and PALLD also had high accuracy in classifying heart failure samples, as evidenced by AUC >0.7 (Figure 6C). Moreover, we found that the expressions of CALU and PALLD were significantly negative correlated with DC, NK CD56dim cells, T cells, Tfh, Tgd, and Th2 cells, and positive correlated with Tcm (Figure 6D).

To further evaluate the value of CALU and PALLD as biomarkers, the levels of CALU and PALLD were assessed in PBMCs isolated from CM and NF patients. In agreement with the results in GSE116250 and GSE5406, the levels of CALU and PALLD were also significantly lower in PBMCs collected from CM patients compared to NF ones (Figure 6E) Collectively, these results suggest that CALU and PALLD may be potential diagnostic biomarkers for heart failure due to ischemic cardiomyopathy.

## Discussion

The prevalence of HF is increasing worldwide and has reached epidemic proportions. Although significant progress has been made in the medications and interventions for HF, the mortality and hospitalization rates remain high (20). Recently, immune cell infiltration has been confirmed to play a vital role in the occurrence and development of cardiovascular diseases (10, 13). Cardiac inflammation and subsequent tissue damage are orchestrated by the infiltration and activation of various immune cells in the myocardium which result in heart failure eventually (20). Thus, it is essential to comprehensively investigate the contributions of infiltrated immune cells in HF. In this study, we analyzed the immune infiltration profiles of CM and NF samples, and identified CALU and PALLD as potential diagnostic biomarkers for heart failure due to ischemic cardiomyopathy by using integrated bioinformatics analyses.

Consistent with a previous report (21), we found that DEGs between NF and CM were mainly enriched into biological processes and pathways related to protein processing and ECM. Structural changes occur in the level of ER and UPR components in cardiomyocytes of patients with HF (22). When maladaptive UPR fails to restore ER homeostasis, it might induce risk factors for HF including increased reactive oxygen species (ROS) production, inflammation and apoptosis which further aggravate HF (22). In addition, numerous studies have explored the relationship between ECM and heart diseases (23, 24). Alterations in the architecture, composition, and distribution of interstitial ECM play a major role in pathological myocardial structural remodeling and left ventricular diastolic dysfunction (25, 26). Myocardial fibrosis is characterized by accumulation of collagen-rich ECM, such as collagen type I and III fibers, results from the predominance of fiber formation and deposition over its degradation and removal (27). ECM dyshomeostasis is also postulated to occur during the development and progression of HF, including changes in the synthesis, processing, degradation, and turnover of proteins such as fibrillar collagen (28). Therefore, those DEGs we found may regulate HF through protein processing and ECM.

Increasing evidence show that immune cell infiltration in the myocardium has adverse effect on heart function (29–31). Single-cell sequencing analyses reveal that the immune cell profiles are remarkably different in healthy and diseased hearts (15, 32, 33). In this study, we found that the abundance of DC, mast cells, NK CD56dim cells, T cells, Tfh, Tgd, and Th2 cells were higher, while the infiltration of eosinophils and Tcm were lower in HF samples, indicating their important roles in the etiology of HF. Consistent with our findings, Patella V et al. and Abdolmaleki F et al. found an increase in numbers of mast cells and T cells in HF, respectively (34, 35). Mast cells initiate adverse myocardial remodeling by activating matrix metalloproteinase (MMP) and fibrosis in the heart (36). Moreover, the profibrotic and antiangiogenic functions of Th17, Th2, and dysfunctional Treg cells are indispensable for the progression to ischemic heart failure (37, 38). On the contrary, inducible depletion of eosinophils exacerbates cardiac dysfunction, cell death, and fibrosis, by producing IL-4 and cationic protein mEar1, fibroblast activation, and neutrophil adhesion (39). Studies from our and other groups suggest a critical role of immune infiltration in the development of heart failure, and further understandings of the effect of each type of infiltrated immune cells may provide us clues for developing novel therapeutic strategies for heart failure.

PALLD encodes a cytoskeleton protein involved in actin reorganization (40), which plays an important role in heart development (41). PALLD was reported to be related to vein graft stenosis after coronary artery bypass grafting. A single-nucleotide polymorphism (SNP) in the PALLD has been reported to be associated with coronary heart disease (CHD) (42). CALU produces a Ca^2+^-binding protein that is localized in the endoplasmic reticulum (ER) (43). During the excitation-contraction coupling process, CALU regulates Ca^2+^ uptake and plays an important role in maintaining normal heart function (44). Our analyses show that the expressions of CALU and PALLD were significantly negative correlated with the abundances of DC, NK CD56dim cells, T cells, Tfh, Tgd and Th2 cells, and Tcm, suggesting that CALU and PALLD may regulate HF via immune-related pathways mediated by the infiltration of these immune cells. More importantly, we confirmed that the expression levels of CALU and PALLD were markedly lower in PBMCs from CM patients compared with NF ones. Collectively, these findings indicate that CALU and PALLD are potential biomarkers for the diagnosis of HF.

Several limitations of the present study should be noted. Firstly, the study was retrospective, further prospective studies are needed to assess the diagnostic and prognostic value of CALU and PALLD in HF. Secondly, the relationships between the biomarkers and immune regulation in heart failure were only verified by assessing their levels in PBMCs from CM and NF patients. Further *in vitro* and *in vivo* experiments are required to explore the detailed mechanisms through which CALU and PALLD regulate inflammatory responses in HF. Taken together, our analyses delineate the potential etiology of HF due to CM and identified immune infiltration related biomarkers in HF, which may provide guidance for diagnosis and treatment of patients with HF.

## Data Availability Statement

The datasets presented in this study can be found in online repositories. The names of the repository/repositories and accession number(s) can be found in the article/Supplementary Material.

## Ethics Statement

The studies involving human participants were reviewed and approved by the Institutional Review Board of the Third Affiliated Hospital, Sun Yat-sen University (IRB: 202102-201-01). The patients/participants provided their written informed consent to participate in this study.

## Author Contributions

YZ and LP contributed to the study concepts and study design and helped in revising the manuscript. XL, SX, and YL drafted the manuscript, performed data management, and bioinformatics analysis. QC were responsible for clinical sample collection. All authors were involved in reporting the results of this study and approved the final version of the submitted manuscript.

## Funding

This work was supported by the National Natural Science Foundation of China (82000466), the Medical Science and Technology Research Project of Guangzhou (Grant No. 202002020030), and the cultivation project of National Natural Science Foundation of the Third Affiliated Hospital, Sun Yat-sen University (Grant No. 2021G2RPYQN11).

## Conflict of Interest

The authors declare that the research was conducted in the absence of any commercial or financial relationships that could be construed as a potential conflict of interest.

## Publisher's Note

All claims expressed in this article are solely those of the authors and do not necessarily represent those of their affiliated organizations, or those of the publisher, the editors and the reviewers. Any product that may be evaluated in this article, or claim that may be made by its manufacturer, is not guaranteed or endorsed by the publisher.
