# Supplementary material for: Identification of CALU and PALLD as Potential Biomarkers Associated With Immune Infiltration in Heart Failure
Source: Front Cardiovasc Med. 2021 Dec 1;8:774755. doi: 10.3389/fcvm.2021.774755 (PMC8671636; doi:10.3389/fcvm.2021.774755)
Supplement: Supplementary file 1 [file Data_Sheet_1.PDF]

# *Supplementary Material*

**Supplementary figure legend**

**Figure S1. Sample clustering dendrogram**. All the samples in GSE5406 dataset are hierarchically clustered and outlier samples are eliminated for WGCNA.

**Figure S2. Sample dendrogram and infiltrated immune cell trait heatmap.** After elimination of outlier samples, all the samples are hierarchically clustered. Infiltrated immune cells are presented as color-coded blocks.

**Figure S3. Screening for the hub module**. (**A**) Determination of the soft-thresholding power for the optimal scale-free topology fit index (scale-free $R^2$) (left) and mean connectivity (right). The optimal soft threshold power was 9 and the red horizontal line represents $R^2 = 0.85$. (**B**) Gene dendrogram and color module. The dendrogram represents the gene clustering. After dynamic tree cut and merging similar modules, eight modules from the co-expression network were identified.
