# Supplementary figures and images for: Identification of CALU and PALLD as Potential Biomarkers Associated With Immune Infiltration in Heart Failure
Source: Front Cardiovasc Med. 2021 Dec 1;8:774755. doi: 10.3389/fcvm.2021.774755 (PMC8671636; doi:10.3389/fcvm.2021.774755)

# Height

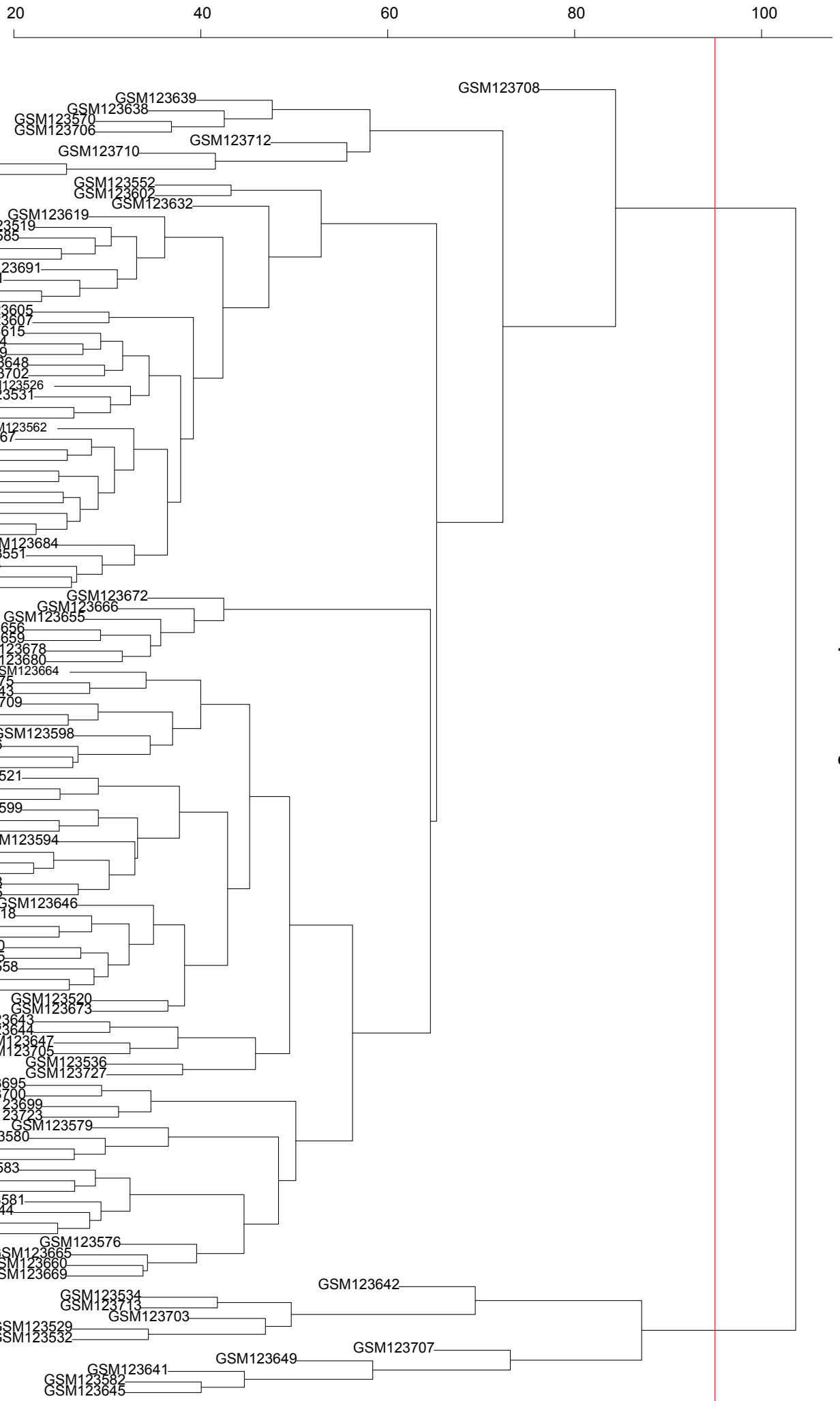

Supplement: Supplementary file 2 [file Data_Sheet_2.PDF]

Sample dendrogram and trait heatmap

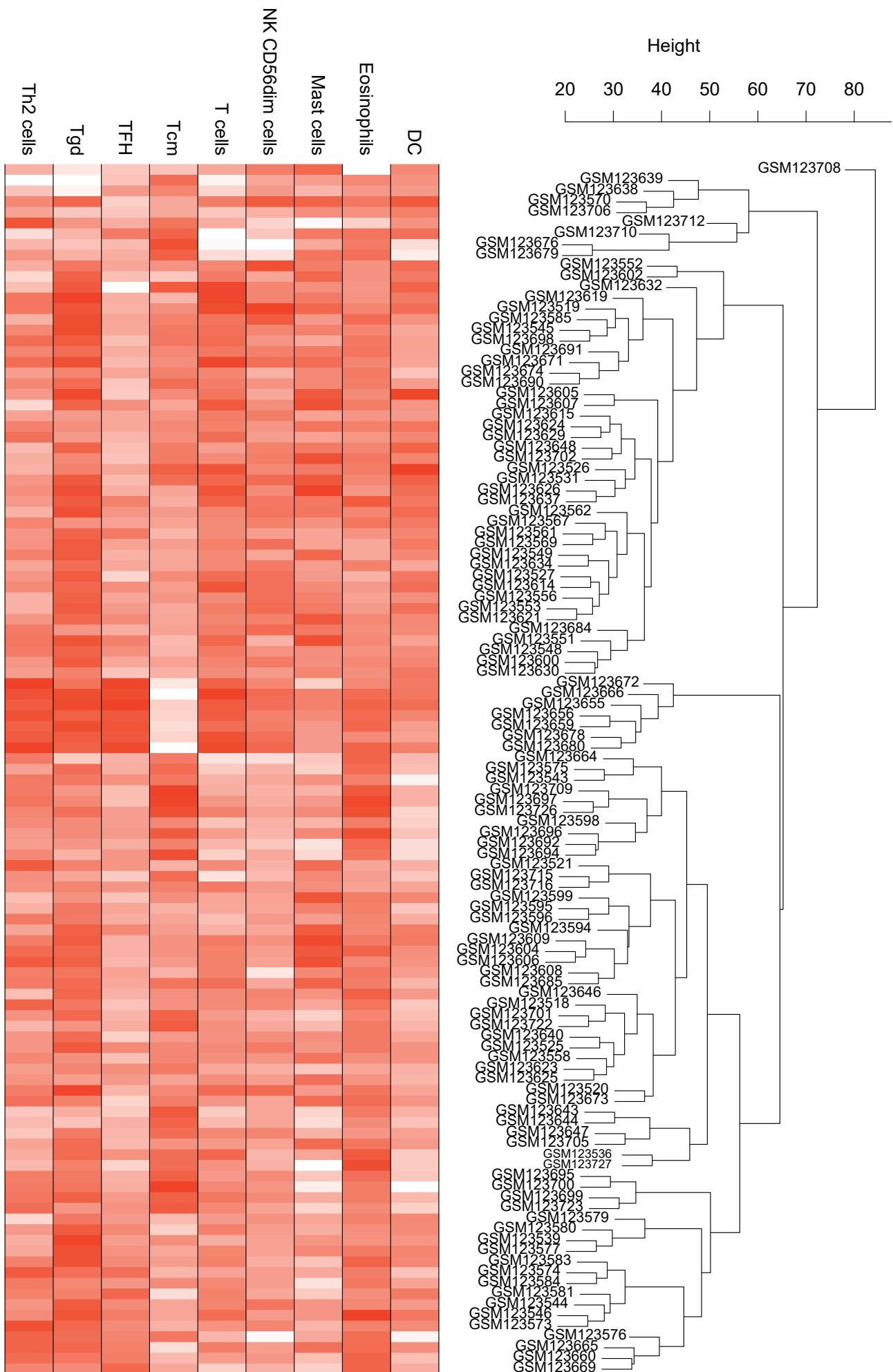

Supplement: Supplementary file 3 [file Data_Sheet_3.PDF]

A.

### Scale independence

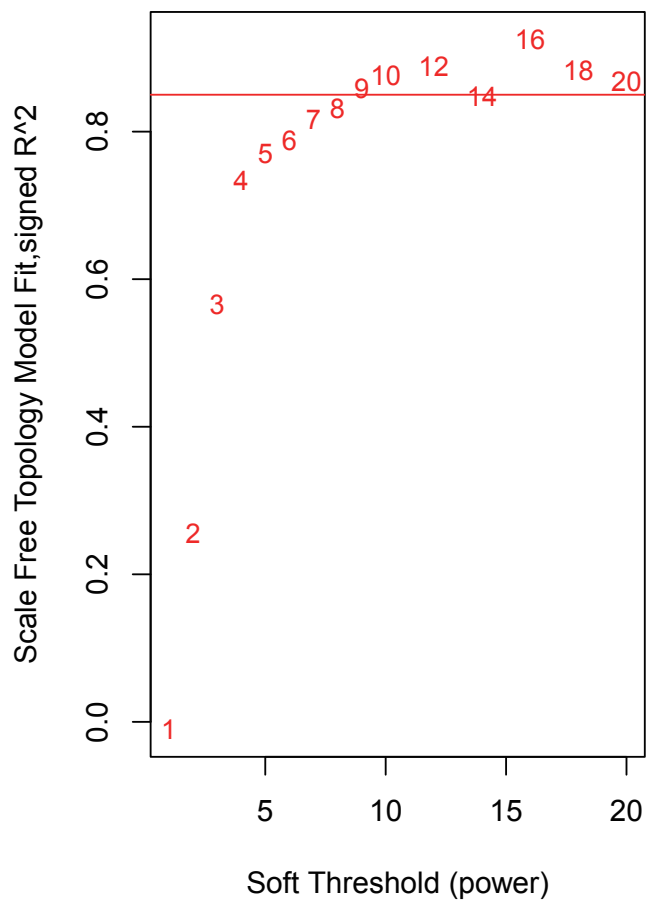

### Mean connectivity

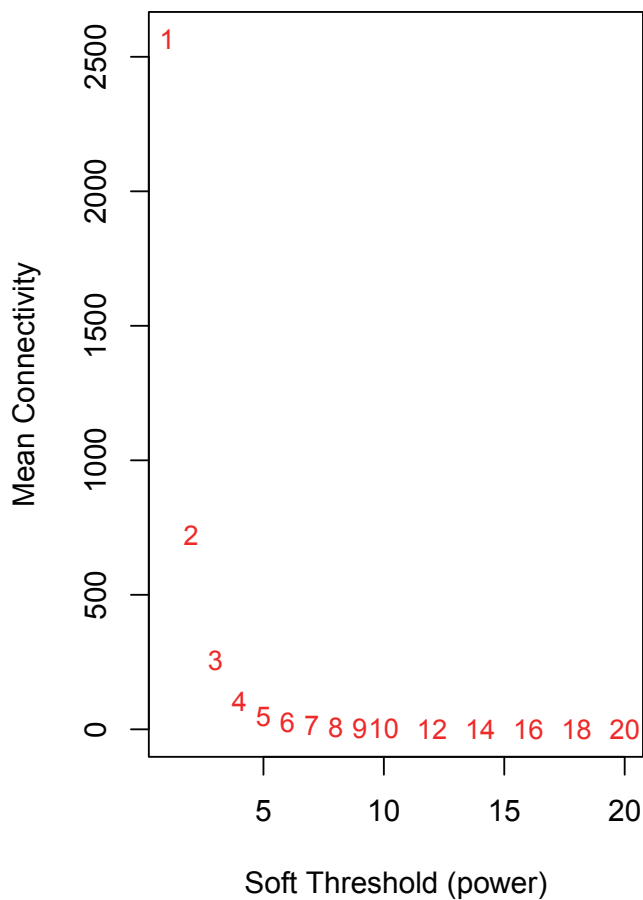

B.

### Gene dendrogram and module colors

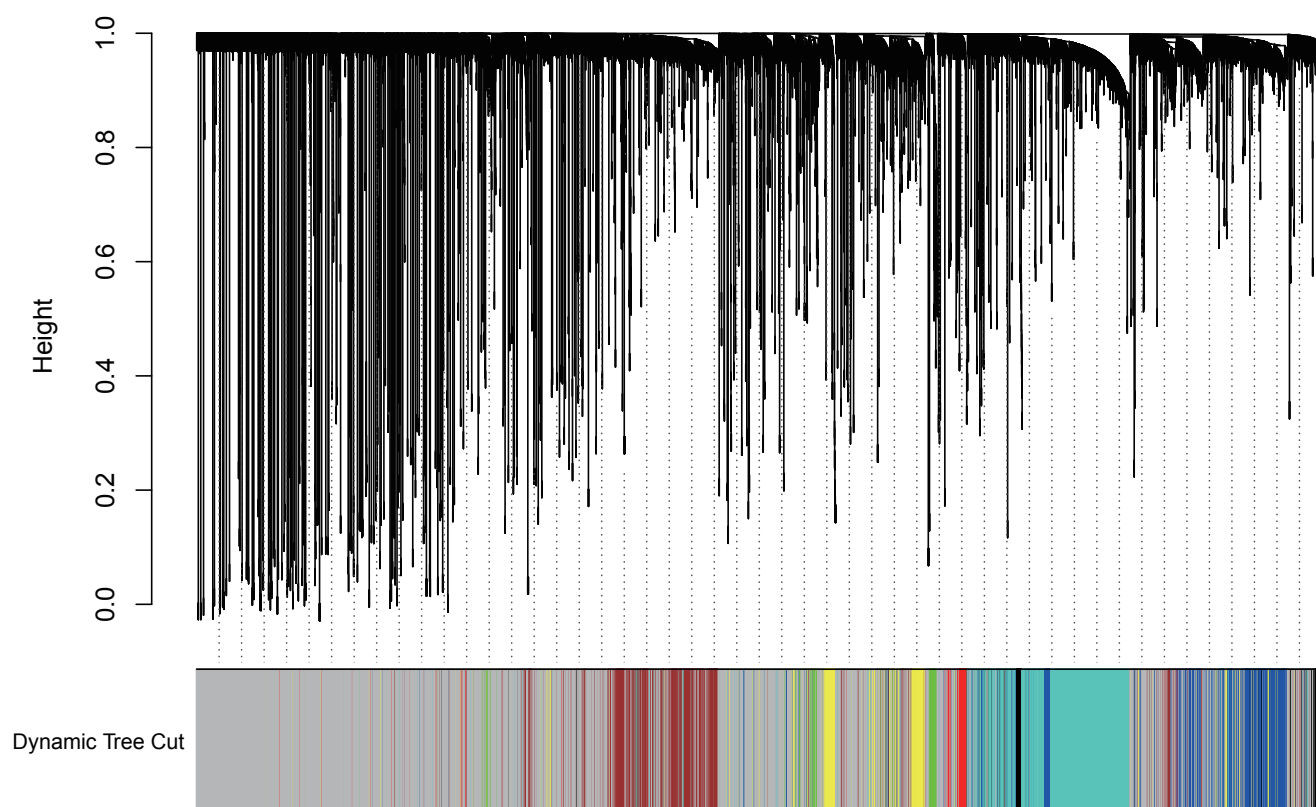

Supplement: Supplementary file 4 [file Data_Sheet_4.PDF]
