# Supplementary material for: Identification of CALU and PALLD as Potential Biomarkers Associated With Immune Infiltration in Heart Failure
Source: Front Cardiovasc Med. 2021 Dec 1;8:774755. doi: 10.3389/fcvm.2021.774755 (PMC8671636; doi:10.3389/fcvm.2021.774755)
Supplement: Supplementary file 7 [file Table_3.DOCX]

**Table 3S.** **The characteristics of included patients**

|  | NF (n=10) | CM (n=10) | *P* value |
| --- | --- | --- | --- |
| Age, years | 67.80±9.05 | 71.50±9.94 | 0.396 |
| BMI, kg/m^2^ | 25.25±4.28 | 24.69±1.98 | 0.71 |
| Systolic blood pressure, mmHg | 133.90±17.72 | 132.00±19.79 | 0.824 |
| Diastolic blood pressure, mmHg | 79.30±4.95 | 87.20±12.71 | 0.84 |
| Heart rate, bpm | 75.00±11.75 | 82.30±12.63 | 0.197 |
| Glucose,mM | 5.45±0.48 | 7.54±4.66 | 0.174 |
| Total cholesterol, mM | 5.37±1.05 | 5.00±1.40 | 0.519 |
| Triglyceride, mM | 1.76±0.76 | 1.42±0.68 | 0.306 |
| HDL-C, mM | 1.17±0.28 | 0.99±0.29 | 0.173 |
| LDL-C, mM | 3.06±0.92 | 3.28±1.1 | 0.629 |
| Scr, μM | 83.23±36.21 | 112.25±54.92 | 0.180 |
| Uric acid, mM | 380.90±99.83 | 442.80±75.24 | 0.135 |
| LVEF, % | 63.40±14.95 | 33.20±6.87 | 0.000 |

Continuous variables are presented as mean ± SD. BMI=body mass index; HDL-C=high-density lipoprotein cholesterol; LDL-C=low-density lipoprotein cholesterol; Scr=serum creatinine; LVEF=left ventricular ejection fraction.
